# Minimal change disease with papillary thyroid carcinoma: a report of two adult cases

**DOI:** 10.1186/s12882-023-03252-9

**Published:** 2023-07-05

**Authors:** Juanjuan Yin, Zexuan Li, Zedong Hao, Zhuanzhuan Yu, Weimin Yu, Guang Yang, Xiaojun Ren

**Affiliations:** 1 Department of Nephrology, Third Hospital of Shanxi Medical University, Shanxi Bethune Hospital, Shanxi Academy of Medical Sciences, Tongji Shanxi Hospital, Taiyuan, 030032 China; 2 Department of General Surgery, the First People’s Hospital of Jinzhong, Jinzhong, 030699 China; 3 Department of Pathology, Shanxi Bethune Hospital, Shanxi Academy of Medical Sciences, Tongji Shanxi Hospital, Third Hospital of Shanxi Medical University, Taiyuan, 030032 China; 4 Taiyuan Kingmed Clinical Laboratory Co., Ltd, Taiyuan, China

**Keywords:** Papillary thyroid carcinoma, Hypothyroidism, Nephrotic syndrome, Minimal change disease

## Abstract

**Background:**

Minimal change disease (MCD), a pathological type of nephrotic syndrome (NS), can occur in patients with tumors. We report two adult cases of MCD associated with papillary thyroid carcinoma (PTC), known to be extremely rare in adults.

**Case presentation:**

A 35-year-old female patient was simultaneously diagnosed with MCD and PTC. The MCD was effectively treated with thyroidectomy and prednisone.In addition, a 50-year-old male patient, who had been diagnosed with PTC three years prior, had MCD confirmed by renal biopsy. The patient achieved complete remission following treatment with tacrolimus and rituximab.

**Conclusions:**

The present case report describes and discusses the diagnostic and treatment processes employed in these two patients. Clinicians need to be aware of the renal effects of treating patients with solid tumors.

## Background

Minimal change disease (MCD) is the primary underlying cause of nephrotic syndrome (NS) in children, but it also accounts for 10–15% of NS in adults [1]. The etiology of MCD is divided into primary and secondary, and common secondary causes include infection, drugs, allergies, and malignancies, the most prevalent of which is Hodgkin’s lymphoma [2]. However, the case of MCD complicated by papillary thyroid carcinoma (PTC) is exceedingly rare, Herein, we report two cases of MCD complicated by PTC in adults and review the relevant literature.

## Case presentation

Patient 1, a previously healthy 35-year-old female, was hospitalized with a two-month history of proteinuria. On physical examination, multiple soybean-sized soft and movable lymph nodes without tenderness or adhesion could be palpated in the neck. There were no positive symptoms in the heart, lungs, or abdomen and no edema in the lower limbs. Laboratory tests revealed nephrotic-range proteinuria (5.02 g /24 h), hypoproteinemia (serum albumin, 28.4 g/L), and hyperlipidemia (serum total cholesterol, 8.02 mmol/L). Thyroid function tests revealed the presence of anti-thyroid peroxidase antibody (186.9 IU/mL) and thyroglobulin antibody (4.3 IU/mL). Table 1 summarizes the laboratory test results of urinalysis and blood serum. Electrocardiography, echocardiography, and chest radiography were all normal. Ultrasonography of the thyroid and cervical lymph nodes showed solid nodules with calcification in the middle of the left lobe of the thyroid (TI-RADS 4b), multiple nodules in the bilateral lobes of the rest of the thyroid (TI-RADS 3), and multiple bilateral cervical lymph nodes.

**Table 1:** Summary of previous reports of patients with thyroid carcinoma and nephrotic syndrome

Author/ reference	Sex	Age	TC	Renal pathology	Time of NS (before or after TC)	Treatment		NS response			
						Carcinoma	NS				
Koopman et al. [4]	F	52	MTC	AAG	After/7years	Surgery, radiation	ACEI	PR			
Han et al. [5]	F	44	PTC	MPGN	Concidence	Surgery	Steroids	CR			
Pattanashetti et al. [6]	M	14	PTC	MPGN	Concidence	Surgery	ACEI	CR			
Liu et al. [8]	M	11	PTC	MCD	Before/80days	Surgery	Steroids	CR			
Tabar et al. [7]	F	21	PTC	FSGS	Before/13months	Surgery radiation	Steroids hemodialysis	Follow up			
Cai et al. [9]	F	38	PTC	MCD	Concidence	Surgery		CR			
Yang et al. [11]	M	56	PTC	NS	After/2years	Lenvatinib(20 mg/day) to sorafenib(400 mg/day)		PR			
Present patient 1	F	35	PTC	MCD	Concidence	surgery, radiation	Steroids	CR			
Present patient 2	M	50	PTC	MCD	After/3years	Surgery, radiation	Tacrolimus, riruximab	CR			

Abbreviations: AAG, amyloid-associated glomerulopathy; ACEI, angiotensin-convertingenzyme inhibitor; CR, complete remission; F, Female; FSGS, focal segmental glomerulosclerosis; M, Male; MCD, minimal change disease; MPGN, membranoproliferative glomerulonephritis; MTC, medullary thyroid carcinoma; PR, partial remission; PTC, papillary thyroid carcinoma; thyroid carcinoma, TC

**Fig. 1:**
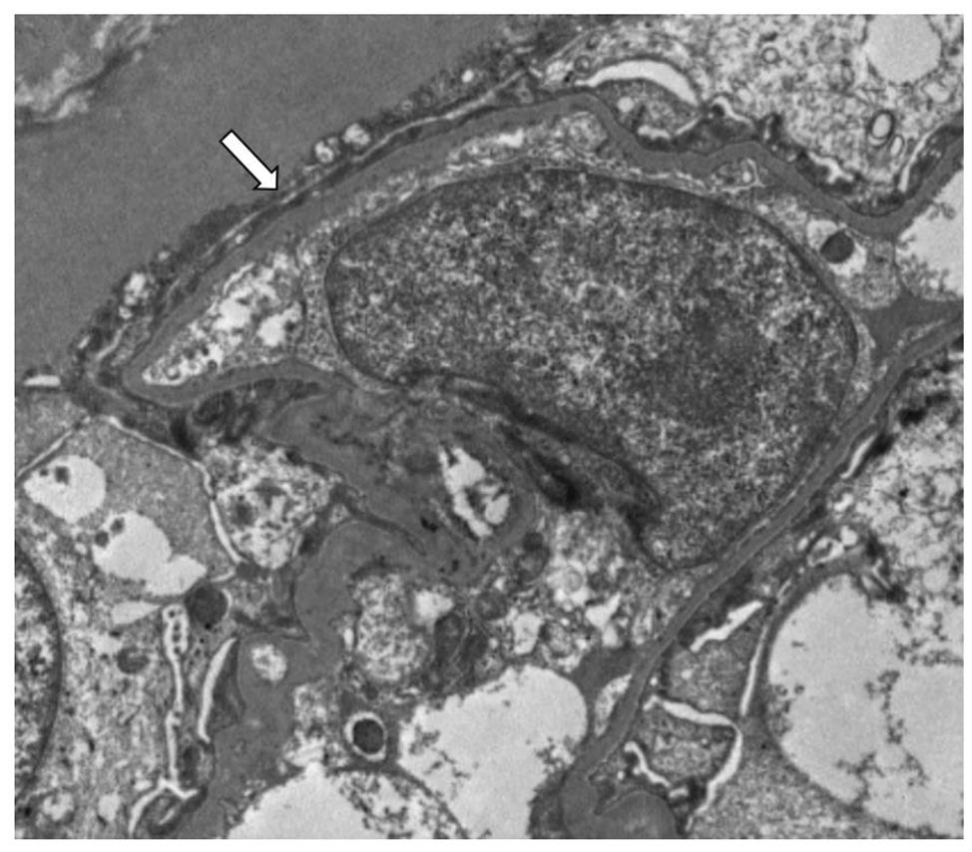
Representative electron micrograph obtained from a kidney biopsy of patient 1. The electron micrograph show diffuse podocyte foot-process effacement (white arrow) without any electron-dense deposition (original magnification ×8000)

**Fig. 2:**
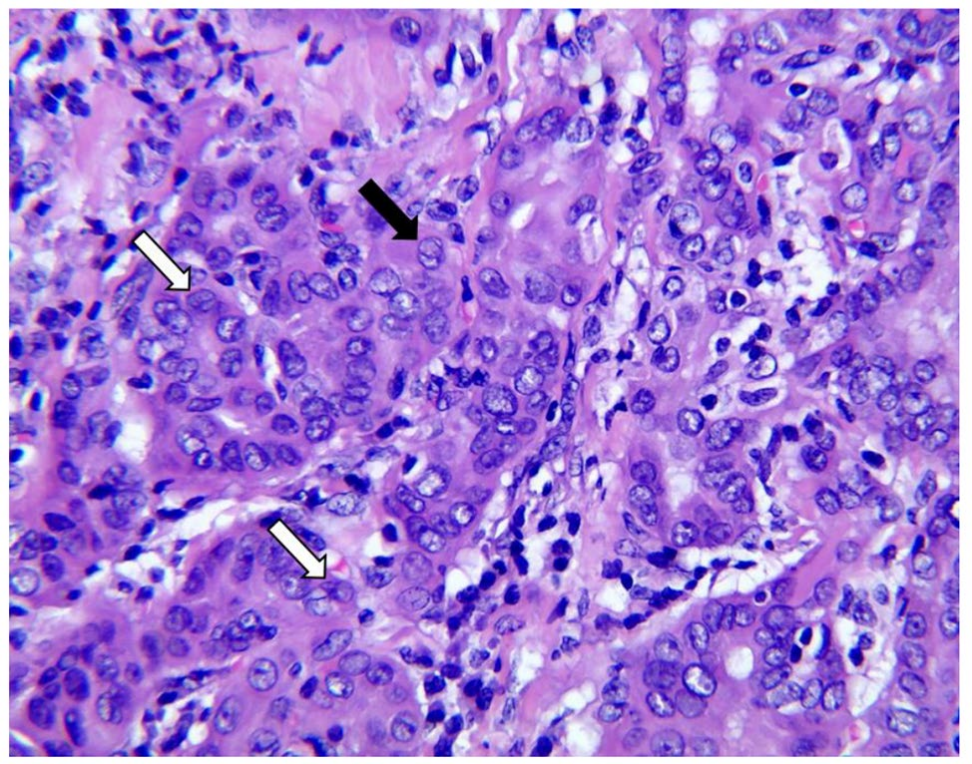
Representative hematoxylin and eosin-stained images of papillary carcinoma of the left thyroid lobe (patient 1). Images show infiltrative growth of tumor cells with enlarged nuclei and ground-glass chromatin. In the nucleus, furrows (white arrows) and false inclusions (black arrows) can be observed (original magnification ×400)

A renal biopsy was performed. Light microscopy revealed 30 glomeruli: one was ischemic sclerotic, while the rest had no obvious mesangial proliferation or basement membrane thickening. Renal tubular epithelial cells were vacuolated and displayed granular degeneration with a few protein tubules in the tubular lumen. Based on immunofluorescence assessment, immunoglobulin(Ig)M, IgG, IgA, C3, Clq, FRA, κ, and λ were negative. Electron microscopy revealed diffuse foot process effacement without immune-type electron-dense deposition in the glomeruli (Fig. 1). Overall, the findings of the biopsied specimen were consistent with MCD. One week after renal biopsy, the patient underwent extended radical resection for thyroid carcinoma, including total thyroidectomy, central lymph node resection, and bilateral cervical lymph node resection during the same admission. Considering the postoperative pathological results, the left thyroid lobe exhibited papillary carcinoma (Fig. 2), the right lobe showed tiny papillary carcinoma, and the lymph nodes presented with metastatic carcinoma (3/8).

After thyroidectomy, the patient was initiated on levothyroxine (112.5 µg daily) and prednisone (40 mg daily). Following a six-week prednisone course, proteinuria decreased (0.13 g/24 h) and serum albumin increased (40.9 g/L) after taking prednisone for 6 weeks. Prednisone was slowly reduced by 5 mg per month, with no relapse during reduction. At the last, follow-up, laboratory tests revealed a thyroid-stimulating hormone (TSH) level of 0.02 mIU/L and proteinuria of 0.16 g/24 h, with oral levothyroxine (112.5 µg) and prednisone (10 mg) administered daily.

Patient 2 was a 50-year-old male who had undergone thyroidectomy of the right lobe and isthmus, right central lymph node dissection of the neck, and radioactive iodine-131 therapy for PTC three years pior. Levothyroxine 175 µg was orally administered daily post-surgery. The patient was hospitalized for two weeks for bilateral lower extremity edema. Laboratory tests revealed considerable proteinuria (21.01 g/24 h), hypoproteinemia (serum albumin, 27 g/L), and hypercholesterolemia (serum total cholesterol, 7.73 mmol/L), resulting in a diagnosis of NS. Based on the thyroid function test results, the patient had hypothyroidism, presenting a TSH level of 266.62 IU/mL, free thyroxine level of 0.51 ng/dL, and thyroglobulin level of 0.04 ng/mL, Additionally, sinus bradycardia (heart rate, 58 beats per minute) was detected by electrocardiography, while echocardiography revealed left atrial enlargement. Thyroid ultrasound showed postoperative thyroidectomy of the right lobe and isthmus, with no substantial local abnormalities.

**Fig. 3:**
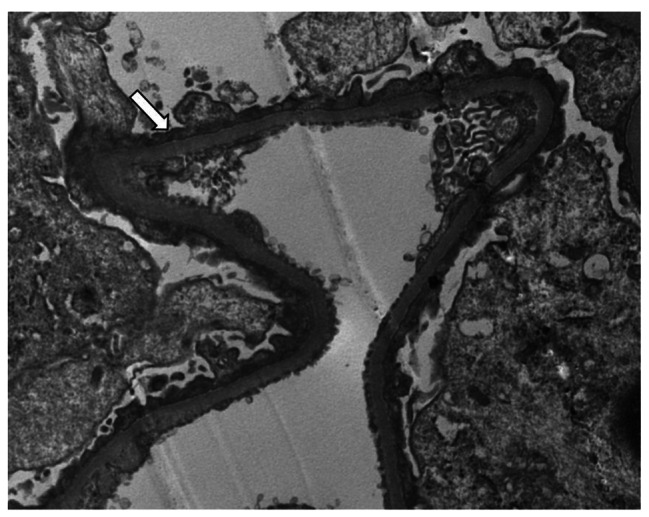
Representative electron micrograph obtained from a kidney biopsy of patient 2. The electron micrograph shows effacement of the podocyte foot process (white arrow) without evidence of electron-dense deposition (original magnification ×6000)

A renal biopsy was performed, and light microscopy revealed 29 glomeruli without focal or increased mesangial matrix or cellularity. Renal tubular epithelial cells were vacuolated and showed granular degeneration. Immunofluorescence microscopy (8 glomeruli present) showed no positive staining. Electron microscopy showed diffuse foot process effacement and no immune-type electron-dense deposits, these findings were consistent with those of MCD (Fig. 3).

The patient refused steroids therapy owing to well-known side effects. Two months after receiving tacrolimus treatment (4 mg/day), the proteinuria decreased from 21.01 to 0.43 g/24 h. After continued tacrolimus treatment for five months, urinalysis revealed worsening proteinuria (1.02 g/24 h). Following a single rituximab(1000 mg intravenous drip) administration, proteinuria was reduced to 0.19 g/24 h upon reevaluation four weeks later. At the time of the last checkup, the patient was taking oral 125 µg of levothyroxine and 1 mg of tacrolimus daily, and proteinuria and TSH levels were 0.16 g/24 h and 0.02 mIU/L,respectively.

## Discussion and conclusions

NS is a clinical syndrome of glomerular diseases with various etiologies and pathological changes, manifested as prototypical symptoms of proteinuria, hypoproteinemia, edema, and hyperlipidemia [3]. It is well-established that the main secondary cause of NS is malignancy. Among malignancies, solid tumors are most commonly associated with membranous nephropathy, and MCD is uncommon. Studies on solid tumors with MCD are only available as case reports, which include cancers of the lungs, colon, kidney, pancreas, bladder, breast, ovary, and renal cells [2]. Considering accumulated literature, only seven cases of thyroid cancer complicated with NS have been reported (Table 1), with pathological types of NS including amyloid-associated glomerulopathy [4], membranoproliferative glomerulonephritis [5, 6], focal segmental glomerulosclerosis [7] and MCD [8, 9]. One case of PTC combined with MCD has been reported in a child [8]. Another case of PTC combined with MCD occurred in an adult who remitted rapidly and completely post-thyroidectomy [9]. Herein, we present two adult patients with MCD and PTC who achieved complete remission following steroids and immunosuppressant therapy.

The precise mechanism underlying the pathogenesis of NS associated with solid tumors remains unclear. Nonetheless, two potential mechanisms have been postulated for the development of MCD in patients with solid tumors. One mechanism may involve the direct infiltration of tumor cells or deposition of tumor metabolites in the glomeruli [8]. For instance, Koopman et al.[4] have described a patient with PTC who developed NS five years post-surgery, had a high plasma calcitonin level, and revealed diffuseglomerular amyloid deposition on renal biopsy. The MTC-induced calcitonin production has been pathologically linked to this deposition. Another mechanism may involve the release of certain other antigens or cytokines from solid tumors, which bind to the glomeruli and result in renal damage [8]. Han et al. [5] and Pattanashetti et al. [6] have described patients with PTC combined with membranoproliferative glomerulonephritis. Both renal tissues displayed considerable electron-dense deposits, potentially resulting in glomerular injury through complement activation, inflammation, and reactive oxygen species production. Furthermore, Taniguchi et al.[10] have described a patient with MCD and rectal cancer. Vascular endothelial cell growth factor (VEGF) is strongly expressed in tumor cells. Following tumor excision, proteinuria disappeared, and VEGF levels returned to normal. VEGF overexpression in tumors has been shown to alter glomerular permeability and glomerular endothelial cell function as well as stimulate podocyte foot process effacement. The occurrence of renal injury six months before or after the tumor should be regarded as tumor-related renal damage [2]. Herein, the first patient had simultaneous MCD and PTC, with no clinical electrolyte disturbances or tumor cell infiltration on assessing renal histopathology. Accordingly, PTC-related immunological dysfunction capable of inducing podocyte injury may contribute to the development of MCD.

In addition to the tumor itself, treatment of the tumor may also be responsible for inducing NS in patients. Yang et al. [11] have reported a patient with PTC who developed NS after lenvatinib treatment. The second patient reported in the present study could represent one such case. Three years prior, the patient had undergone thyroidectomy and radioactive iodine-131 therapy for PTC. After hospitalization, the patient was diagnosed with concurrent MCD and hypothyroidism. The emergence of MCD in this patient could be attributed to hypothyroidism following PTC therapy. given that the onset of PTC and MCD occurred three years apart. Previous studies have shown that MCD is a prominent pathology in patients with hypothyroidism [12], as hypothyroidism can affects renal function, directly or indirectly. On the one hand, hypothyroidism increases the expression of thyroid receptor-1 on podocytes, inducing cytoskeletal rearrangement and podocyte foot process effacement, which in turn leads to proteinuria [13]. Conversely, thyroxine has positive time-varying and force-varying cardiac effects. Hypothyroidism weakens these effects, resulting in a reduce cardiac output and renal blood flow, indirectly damaging renal tissue [14]. Meanwhile, NS may also induce hypothyroidism owing to the loss of hormone-binding proteins. A large fraction of plasma protein-bound thyroid hormones can evade the glomerular filtration barrier and are lost in the urine, as they only undergo partial reabsorption in the proximal tubule by megalin and cubilin complexes.A vicious circle could exist between hypothyroidism and NS [15]. The second patient in this report had normal echocardiography and electrocardiography findings prior to the diagnosis of PTC, but sinus bradycardia and left atrial enlargement were found when MCD was diagnosed. Therefore,we speculate that NS may aggravate hypothyroidism in the patient.

In summary, we presented case reports describing patients with MCD complicated by PTC. In one patient, MCD may be attributed to renal damage due to PTC itself; in the second patient, MCD may be caused by hypothyroidism following PTC treatment. Our report suggests that, clinicians need to remain highly aware of any potential renal adverse effects when treating patients with solid tumors. If patients with solid tumors exhibit renal damage, the underlying cause should be investigated to improve the prognosis.

## Data Availability

Not applicable.

## Abbreviations

Ig: immunoglobulin
MCD: minimal change disease
NS: nephrotic syndrome
PTC: papillary thyroid carcinoma
TSH: thyroid-stimulating hormone
VEGF: vascular endothelial cell growth factor
